# The Effect of Acidifier-Dextrose against Hen Day Production and Feed Conversion Ratio in Laying Hens Infected with Avian Pathogenic *Escherichia coli*

**DOI:** 10.1155/2021/6610778

**Published:** 2021-03-26

**Authors:** Sunaryo Hadi Warsito, Emy Koestanti Sabdoningrum, Nana Tripalupi, Ayu Nur Hidayati, Anwar Ma'ruf, Poedji Hastutiek, Mirni Lamid, M. Anam Al-Arif, Herry Agoes Hermadi, Oky Setyo Widodo

**Affiliations:** ^1^Department of Animal Husbandry, Faculty of Veterinary Medicine, Universitas Airlangga, Surabaya, Indonesia; ^2^Faculty of Veterinary Medicine, Universitas Airlangga, Surabaya, Indonesia; ^3^Faculty of Vocational, Universitas Airlangga, Surabaya, Indonesia; ^4^Department of Parasitology, Faculty of Veterinary Medicine, Universitas Airlangga, Surabaya, Indonesia; ^5^Department of Veterinary Reproduction, Faculty of Veterinary Medicine, Universitas Airlangga, Surabaya, Indonesia

## Abstract

Colibacillosis in Indonesia until now still appears frequently, so the case of colibacillosis laying hens cannot reach the peak of egg production; the egg production period is delayed and easily infected with other diseases. The purpose of this research is that the acidifier-dextrose combination is expected to be able to suppress the development of Avian Pathogenic *Escherichia coli* (APEC) bacteria in laying hens so that, in the end, the case of colibacillosis can be controlled in Indonesia. A total of 240 heads of laying hens were divided into 6 treatments and each consisted of 40 replications. The results of this research state that a combination of acidifier-dextrose can increase Hen Day Production (*p* < 0.05) and decrease Feed Conversion Ratio (*p* < 0.05) in laying hens infected with APEC. The Hen Day Production results of the treatment group infected with APEC showed the lowest results, amounting to 65.75% whereas the other treatments are still above 90%. Furthermore, the highest Feed Conversion Ratio results were on treatments infected with APEC, which amounted to 2.17 while other treatments of the Feed Conversion Ratio results are still below 1.80. In general, the use of a combination of acidifier and dextrose with the lowest dose, that is, 1 g/3.75 liters of drinking water can still give good results to Hen Day Production and Feed Conversion Ratio for laying hens infected with APEC. Giving combination of acidifier-dextrose can increase Hen Day Production and decrease Feed Conversion Ratio in laying hens infected with APEC. The recommended dosage of acidifier-dextrose combination in laying hens based on this research is 1 g/3.75 liters of drinking water.

## 1. Introduction

This research was inspired by the frequent cases of colibacillosis in laying hens in the field in Indonesia where the economic calculation of the total loss due to colibacillosis reached 13.10% of the total assets of poultry in Indonesia [1].

The research aims to know the effect of the combination of acidifier and dextrose on the Hen Day Production (HDP) and Feed Conversion Ratio (FCR) of laying hens infected with Avian Pathogenic *Escherichia coli* (APEC). Decreased egg production in laying chickens can occur, one of which is caused by colibacillosis. According to [2], the etiology of colibacillosis can be either due to primary infection with APEC or secondary (opportunistic) infection after a primary insult has occurred. *E. coli* are Gram-negative, rod-shaped bacteria considered normal inhabitants of the avian digestive tract. While most strains are considered to be nonpathogenic, certain strains have the ability to cause clinical disease. Pathogenic strains are commonly of the O1, O2, and O78 serotypes. Reference [3] states that *Escherichia coli* attacks the reproductive tract so that later it will affect the Hen Day Production (HDP) and Feed Conversion Ratio (FCR).

Therefore, efforts to increase egg production can be done by giving acidifier in the form of citric acid and thus the combination by dextrose. Reference [4] states that acidifier is one of the feed additives that can provide a positive influence in the form of control of microflora in the digestive tract. The acidifier in general can replace the role of antibiotics, increase egg production and egg quality; balance the condition of the digestive tract microflora; and increase the absorption of food juices in the small intestine. The effects of organic acids on intestinal microflora include the following: specific effects of acid anions on cellular enzymes or membranes, internal pH values and buffering capacity of cells, the amount of ATP used in pumping protons, and the transport of acid molecules. In addition, [5] states that the use of citric acid can create an acidic atmosphere of pH 3.5–4.0 in the intestinal tract which inhibits the replication of *Escherichia coli*, *Salmonella* sp., and other Gram-negative bacteria.

Laying hens require nutrition for their survival. The absorption of nutrients will be maximized when the body has good energy reserves. Farmers usually use sugar water as an energy source [6]. Dextrose is the name of simple sugar that is made from corn and is chemically identical to glucose or blood sugar. Dextrose is often used in baking products as a sweetener and can be commonly found in items such as processed foods and corn syrup. Dextrose also has medicinal purposes and is an energy source [7]. Carbohydrates contained in feed additives can increase growth, feed conversion, and egg production in laying hens [8].

Based on existing references, it is clear that colibacillosis can interfere with the reproduction of laying chickens while the use of acidifiers can inhibit the development of bacteria and the use of dextrose is very good as an energy source. Therefore, it is necessary to do research on laying chickens infected by colibacillosis given the acidifier-dextrose combination to determine the real results.

## 2. Materials and Methods

Experimental animals used were ISA Brown strain laying hens aged 26 weeks infected with Avian Pathogenic *Escherichia coli* (APEC) with 6 treatments and 40 replications so that the number of laying chickens in this study was 240 heads of a total population of 10,000 heads. APEC bacterial isolates were obtained from layer farms in the Jabon area of Sidoarjo, East Java, Indonesia, and bacterial propagation was carried out at the Laboratory Center for Infection Special Hospitals at Universitas Airlangga. APEC bacteria grown on EMBA media made suspension and dilution according to McFarland I standard (suspension contains 3 × 10^8^ CFU/ml).

At first, the laying chickens were adapted for 7 days with environmental conditions. Then, on the 8^th^ day, laying chickens were infected with APEC 10^8^ CFU/ml as much as 1 ml/kg body weight orally, and on the next 7 days, symptoms were seen such as pale chickens with dull feathers, chickens rarely lay eggs and when laying eggs, the eggs are of low quality. The laying chickens' morbidity given by APEC was up to 100%, while the mortality was 0%. However, in terms of egg productivity, there was a decrease of 45–55%. Then, on the same day, laying chickens were given a combination of acidifier-dextrose according to the treatment dose in drinking water for 4 weeks.

The treatments given to the group are as follows: P0 is a treatment for laying hens that are not infected with APEC and are given a drink without a combination of acidifier-dextrose, P0(−) is a treatment for laying hens that are not infected with APEC and are given a drink combination of acidifier-dextrose with a dose of 1 g/2.5 liters of drinking water, P0(+) is a treatment for laying hens infected with APEC as much as 2 ml/head orally and given a drink without the combination of acidifier-dextrose, P1 is a treatment for infected laying hens APEC as much as 2 ml/head orally and given a combination of acidifier-dextrose at a dose of 1 g/1.25 liters of drinking water, P2 is the treatment of laying hens infected with APEC as much as 2 ml/head orally and given a combination of acidifier-dextrose at a dose 1 g/2.5 liters of drinking water, and P3 is the treatment of laying hens infected with APEC as much as 2 ml/head orally and given a combination of acidifier-dextrose at a dose of 1 g/3.75 liters of drinking water. Furthermore, the results of the study were analysed by using Analysis of Variance and followed by Duncan's Multiple Range Test.

## 3. Results

### 3.1. Hen Day Production

The results of Hen Day Production (HDP) calculation in laying hens infected with *Escherichia coli* and given a combination of acidifier-dextrose through drinking water with 6 treatments and 40 repetitions after treatment can be seen in Table 1. The HDP results of the treatment group infected with APEC were the lowest, amounting to 65.75% whereas the other treatments are still above 90%.

### 3.2. Feed Conversion Ratio

The results of FCR calculation in laying hens infected with *Escherichia coli* and given a combination of acidifier-dextrose through drinking water with 6 treatments and 40 repetitions after treatment can be seen in Table 1. The highest FCR results were on treatments infected with APEC, which amounted to 2.17 while in other treatments, the FCR results are still below 1.80. In general, the use of a combination of acidifier and dextrose with the lowest dose, that is, 1 g/3.75 liters of drinking water, can still give good results to HDP and FCR for laying hens infected with APEC.

## 4. Discussion

### 4.1. Hen Day Production

The observations showed that laying hens produced the highest HDP at P0, P0(−), P1, P2, and P3 and the lowest was P0(+). This is influenced by the presence of *Escherichia coli* bacteria that attack the reproductive tract in laying hens and without given the combination of acidifier-dextrose. This is consistent with the opinion of [9] that the infection of pathogenic *Escherichia coli* pathogens that attack chickens, namely, *Avian Pathogenic Escherichia coli* (APEC) which attacks the reproductive tract, can affect the production performance.

APEC infection in laying chickens is characterized by salpingitis which causes egg peritonitis if the yolk is stored in the peritoneal cavity [10]. This shows that if yolk remains stored in the peritoneal cavity, the ovary cannot reach the next egg formation process, likewise with [11] statement that *Escherichia coli* bacteria can infect the reproductive organs, especially the infundibulum in laying hens before the egg-laying period. Infundibulum functions to catch mature ovum cells, due to the presence of *Escherichia coli* bacteria that infects the infundibulum so that the chicken cannot produce eggs optimally, so the egg formation process is inhibited and HDP will decrease.

Acidifier-dextrose contained in chicken drinking water in the treatment of P1, P2, and P3 can increase the HDP in chickens infected with *Escherichia coli* because acidifier in the form of citric acid in this research functions to regulate pH in the digestive tract that is able to inhibit the replication of *Escherichia coli* bacteria, besides that dextrose is able to add energy to the development of laying chickens so that the combination of acidifier-dextrose in drinking water can increase HDP even though the chicken is infected with *Escherichia coli*. This is in accordance with the opinion of [12] which states that citric acid is a natural ingredient or synthesis that works to improve the digestibility of feed and maintain microbial balance in the digestive tract through regulating the pH of the digestive tract. The digestive tract which has a low pH will reduce the population of pathogenic bacteria. The use of citric acid creates an acidic pH of 3.5–4.0 in the intestinal tract which inhibits the replication of *Escherichia coli* [5].

Reference [13] states that there are two acid mechanisms in inhibiting bacterial development, namely, the undissociated form of citric acid will directly enter the lipid membrane of gram-negative bacteria and citric acid dissociates directly because of the ability of its hydrogen-free protons which can lower the pH so as to create acidic conditions, so that these conditions have a negative effect on the presence of pathogenic bacteria. Organic acids that enter the cytoplasm which has neutral pH conditions inhibit bacterial growth by inhibiting the oxidative phosphorylation process and increasing energy consumption. Neutral pH conditions must be maintained by the cytoplasm of the cell and for that when the process of transfer of protons from inside and outside the cell to balance the acidity that occurs, it requires energy from cellular ATP which can then cause the energy in the cell to be drained. Energy needs can be fulfilled by the addition of dextrose which is a monosaccharide from the end product of carbohydrate digestion [7]. Carbohydrates contained in dextrose can increase egg production [8]. Reference [14] states that dextrose also has the function of pairing with protein so that a small amount of protein is disassembled to produce energy.

Giving a combination of acidifier-dextrose with different dosages shows a difference in the average HDP yield; more water is used to be leached acidifier-dextrose; and a dose of 1 g/3.75 liters is the best dose to improve the performance of chickens by infected with APEC. This is because the levels of citric acid that are too high in drinking water for laying hens cause the pH to be too acidic so that the host's unfavorable conditions are due to the irritant nature of the citric acid. However, there is a tendency towards a better direction with increasing doses of acidifier-dextrose even though the statistical test treatments (P1, P2, and P3) are not significantly different. This includes that, by adding acidifier-dextrose in laying chickens, drinking water is able to optimize the daily production of eggs (HDP) to remain high in laying hens infected with APEC so that breeders losses due to cases of colibacillosis can be avoided or corrected.

### 4.2. Feed Conversion Ratio

Data shows that the administration of a combination of acidifier-dextrose in laying hens infected with APEC bacteria can affect Feed Conversion Ratio (FCR). Data produced from the lowest to highest are P3 (1.6272), P2 (1.6322), P1 (1.7494), P0 (1.7666), P0(−) (1.7996), and P0(+) (2.1736). The lowest FCR was shown in the P3 group of 1.6272, namely, the group treated with acidifier-dextrose at a dose of 1 g/3.75 liters of drinking water, presumably because in drinking water, there were acidifier-dextrose contents in the appropriate dosage. So, the role of acidifier (citric acid) appears as organic acids in influencing the atmosphere of acidic pH in digestion which in turn can suppress the development of APEC bacteria. This is in accordance with the opinion of [6] which states that the administration of citric acid in appropriate quantities can reduce intestinal pH in the digestive system and can increase enzymatic activity so as to increase absorption of nutrients in feed. Similar results were also obtained by [15] which state that malic acids as organic acid have the potential for reduction of *E. coli* population in chicken intestine in the broiler and layer chickens.

The treatment of P0 was not significantly different from P0(−), whereas P0(+) was significantly different from P1, P2, and P3. It was suspected that healthy laying chickens had not occurred in the growth of intestinal villi because the chickens had entered the egg-laying phase, whereas in laying chickens infected with APEC, acidifier-dextrose works more effectively because the acidifier is able to improve intestinal health by making the pH of the digestive tract become acidic which negatively influences the growth of pathogenic bacteria such as APEC and the function of dextrose which is able to increase stamina in chicken. P2 and P3 treatments both showed good feed conversion rates but P3 tended to be more efficient because the dose used was smaller than P2.

Acidification in drinking water is preferred in the broiler and layer industries to improve production performance [16, 17] and various organic acids have been tested. The use of acidifier-dextrose is the right combination to increase production performance because dextrose also functions as a thermoregulator in addition to adding energy to chickens. This is in accordance with the opinion of [18] which states that dextrose is involved not only in increasing reproduction performance but also in regulation. Poultry that consumes dextrose-containing water is also seen to have lower mortality from heat exposure compared to those who only consume plain water [19].

High feed conversion rates indicate less efficient use of feed, conversely if the feed conversion rate decreases, means that the use of feed is more efficient. P0(+) treatment showed the highest feed conversion rate of 2.1736. This is due to the presence of pathogenic *Escherichia coli* strains that attack the chicken reproductive tract, namely, *Avian Pathogenic Escherichia coli* (APEC). This is consistent with the opinion of [11] said that the *Escherichia coli* bacteria can infect the reproductive organs in laying hens before the period of laying eggs. Further said by [20], the most typical forms of localized infection due to APEC as a primary pathogen are infections of the reproductive tract, omphalitis, and yolk sac infection. Infections of the reproductive tract include salpingitis/peritonitis/Salpingperitonitis Syndrome (SPS), which is very common in laying hens and the *E. coli* Peritonitis Syndrome (EPS) which is more acute and septicaemic than SPS and affects laying chickens from the start of egg production to peak production. The route of infection seems to be of respiratory and vaginal origin.

The addition of a combination of acidifier-dextrose at a dose of 1 g/3.75 liters of drinking water resulted in the lowest FCR and was the best dose for laying hens infected with APEC. The treatments P1, P2, and P3 in the statistical test are not significantly different, but there is a tendency towards a better direction as a decrease in the dose of the combination of acidifier-dextrose at a dose of 1 g/3.75 liters of drinking water, so as to reduce the FCR in laying hens that is infected with APEC. This can minimize the losses of farmers due to cases of colibacillosis.

## 5. Conclusions

Based on the results of research on the effect of giving acidifier-dextrose to HDP and FCR in laying hens infected with APEC, the following can be concluded: 1. hiving acidifier-dextrose combination can increase Hen Day Production (HDP) and reduce the number of Feed Conversion Ratio (FCR) in laying hens infected with APEC; 2. the use of citric acid presumed creates an acidic in the intestinal tract which inhibits the replication of *Escherichia coli* and the use of dextrose because it is a source of energy and is thought to be able to help the condition of weak chickens become more energetic so that they can quickly help the recovery of laying hens affected by APEC; 3. the recommended dosage of acidifier-dextrose combination in laying hens based on this research is 1 g/3.75 liters of drinking water.

## Figures and Tables

**Table 1:** Average HDP and FCR in laying chickens infected with APEC and given a combination of acidifier-dextrose through drinking water.

Treatments	HDP ± SE (%)	FCR ± SE
P0	96.85^b^ ± 5.16	1.77^a^ ± 0.09
P0(−)	98.50^b^ ± 4.74	1.80^a^ ± 0.02
P0(+)	65.75^a^ ± 8.99	2.17^b^ ± 0.55
P1	90.75^b^ ± 9.05	1.75^a^ ± 0.12
P2	95.25^b^ ± 7.08	1.63^a^ ± 0.08
P3	98.25^b^ ± 5.53	1.63^a^ ± 0.04

*Note.* Different superscripts show a real difference (*p* < 0.05).

## Data Availability

The datasets used and analysed during the current study are available from the result of the conducted treatment on laying hens farm in field. The datasets generated and/or analysed during the current study are not publicly available due to a research patent but are available from the corresponding author on reasonable request.

## Abbreviations

APEC: Avian pathogenic *Escherichia coli*
HDP: Hen Day Production
FCR: Feed Conversion Ratio
SE: Standard error
P0: Control
P0(−): Negative control
P0(+): Positive control
P1: 1^st^ treatment
P2: 2^nd^ treatment
P3: 3^rd^ treatment
EMBA: Eosin methylene blue agar
CFU: Colony Forming Unit.
